# Potential of Cricket (*Acheta domesticus*) Flour as a Lean Meat Replacer in the Development of Beef Patties

**DOI:** 10.3390/foods13020286

**Published:** 2024-01-16

**Authors:** Carlos Pasqualin Cavalheiro, Claudia Ruiz-Capillas, Ana M. Herrero, Tatiana Pintado, Camila Cristina Avelar de Sousa, Juliana Sant’Ana Falcão Leite, Maurício Costa Alves da Silva

**Affiliations:** 1 INDMEAT Group, Department of Meat and Fish Products, Institute of Food Science, Technology and Nutrition (ICTAN-CSIC), 28040 Madrid, Spain; ana.herrero@ictan.csic.es (A.M.H.);; 2 Laboratório de Inspeção e Tecnologia de Carnes e Derivados (LabCarne), Escola de Medicina Veterinária e Zootecnia, Universidade Federal da Bahia, Salvador 40170-115, Brazil

**Keywords:** beef patties, cricket flour, cooking, brownish color, texture

## Abstract

This study examined the incorporation of cricket (*Acheta domesticus*) flour (CF) (0, control; 5.0%, CF5.0; 7.5%, CF7.5; and 10.0%, CF10.0) as a lean meat replacer in beef patties and its impact on composition, microbiological, sensory, and technological properties, as well as its influence on the cooking process. The inclusion of CF led to beef patties with significantly higher protein levels than the control group. Additionally, an elevation in total viable (TVC) and lactic acid bacteria (LAB) counts was observed. However, Enterobacteriaceae counts remained at safe levels. CF5.0 demonstrated similar sensory scores and purchase intention to the control treatment. CF7.5 and CF10.0 showed comparable sensory scores to the control except for texture attributes. The inclusion of CF significantly reduced cooking loss and diameter reduction values. Beef patties with CF were notably firmer and had a browner color than the control. In general, the cooking process impacted the technological properties similarly in both the control and beef patties with CF. In all cooked samples, no significant differences in pH, redness (a*), or texture were observed. This study demonstrated that incorporating up to 5.0% CF into beef patties is optimal in terms of composition, technological, sensorial, and cooking properties.

## 1. Introduction

The global population is projected to reach 9 billion people by 2050, resulting in heightened demand for agricultural production, particularly in terms of animal protein [1]. Consequently, alternative protein sources are gaining popularity as consumers seek innovative and eco-friendly options. Algae [2], plants [3], and edible insects [4] can serve as substitutes for lean meat or in combination with lean meat to produce hybrid meat products. Within this context, these newly reformulated meat products must closely resemble traditional meat products in terms of appearance, texture, mouthfeel, flavor, and cookability [5].

For centuries, edible insects have been part of human diets worldwide, with a wide variety of species commonly consumed in many Asian and African countries [6]. Insects offer a valuable source of protein, fatty acids, vitamins, and minerals, making them an excellent nutritional resource [7]. However, they remain relatively unfamiliar in Western countries [8]. According to van Huis and Rumpold [9], a compelling strategy to encourage insect consumption is to incorporate them in a less recognizable form within familiar products and to ensure that insect-based products are delicious. In this context, evaluating the sensory properties becomes crucial to enhance consumer willingness to purchase new insect-based foods, including meat products [10]. Moreover, edible insects possess remarkable water-holding capacity (WHC) and oil-holding capacity (OHC), which are critical attributes for meat applications, especially concerning texture. They also exhibit strong emulsion and gelling properties [11], which are important for the development of new meat products. From a nutritional perspective, incorporating edible insects can enhance the protein content of meat products [12]. Consequently, some studies have already explored the partial replacement of lean meat with super worms (*Zophobas morio*), mealworms (*Tenebrio molitor*), grasshoppers (*Sphenarium purpurascens*), and silkworms (*Bombyx mori*) in emulsified cooked sausages [13,14,15,16].

Recently, the European Commission has granted authorization for the marketing of frozen, dried, and powdered forms of cricket house (*A. domesticus*) as a novel food [17]. In the USA, the use of edible insects as food is regulated by the US Food and Drug Administration (FDA). These edible insects meet standards of cleanliness and health, being free of pathogens and toxins. Additionally, they must have been produced, packaged, stored, and transported under sanitary conditions and must be properly labeled [18,19]. In Canada, they can be sold without specific legislation due to their history of safe use as a food [20,21]. However, in other countries such as Mexico, Australia, New Zealand [19], and Brazil, there are no specific regulations regarding the use of edible insects as food.

However, owing to the growing interest in edible insects, CF is emerging as a promising alternative for the reformulation of meat products due to its high protein content (72%) and its potential to enhance technological properties [22]. Nevertheless, their incorporation into the human diet must consider microbiological, physical, chemical, allergenic, parasitic, and toxicological aspects [23]. Some authors have replaced lean meat with cricket house in emulsified cooked meat products [12,24]. However, to the best of our knowledge, there have been no studies employing CF (*A. domesticus*) in fresh meat products, such as beef patties, which are extremely popular worldwide due to their convenience, affordability, high nutritional value, and sensory satisfaction. Therefore, this study aimed to develop reformulated beef patties with CF at different ratios to replace lean meat. The composition, microbiological, sensory, and technological properties of these beef patties were thoroughly evaluated. Additionally, this study assessed how the cooking process impacted the technological properties of beef patties, as this is a crucial step in the consumption of this type of meat product.

## 2. Materials and Methods

### 2.1. Raw Materials

Cricket (*A. domesticus*) flour (CF) was purchased from the UK market (Derby, UK). According to the producer, it underwent a process of defatting, microwave drying, and milling (120–250 microns). The CF was free from artificial colors, flavorings, preservatives, monosodium glutamate, gluten, and dairy. The composition of the CF had been reported as 6.8% moisture, 66% protein, and 16.27% fats. In addition, the pH was 6.41, and its color parameters were as follows: L* (66.8), a* (3.1), and b* (14.4) [12].

Fresh beef (21.58% protein and 16.66% fat) was purchased from a local slaughterhouse in Madrid (Spain). It was then ground using a 6 mm plate meat grinder, vacuum-packed, and frozen at −20 °C until needed. The additives used for preparing the beef patties included sodium chloride (Panreac Química S. A., Barcelona, Spain) and onion powder (Pilarica, S.A., Paterna, Valencia).

### 2.2. Preparation and Formulation of Beef Patties

Four different formulations of beef patties were prepared. The first treatment, labeled as the control, consisted of 86.5% beef lean meat, 10.0% water, 2.0% onion powder, and 1.5% salt. The other three formulations were manufactured incorporating 5.0% (CF5.0), 7.5% (CF7.5), and 10.0% (CF10.0) of CF, which replaced an equivalent amount of beef lean meat in each respective formulation to ensure a well-mixed blend of all ingredients and the adequate formation of meat mass. This replacement provides an energy content of 20.49, 30.73, and 40.98 kcal/100 g, respectively (calculated based on 9 kcal/g for fat and 4 kcal/g for protein). The selection of the best levels of cricket flour incorporation was determined by taking into account the processing and production conditions, ensuring a thorough mixture of all ingredients, and achieving a suitable final formation of the beef patties. This choice was also influenced by our previous experience with the use of cricket flour in sausages [12]. Additionally, we conducted preliminary sensory sessions to assess the range of cricket flour levels that were more adequate for inclusion in the beef patties. The ground meat and non-meat ingredients were added at different stages and homogenized for a total of 3 min using a mixer (Hobart. Model N50 6, Troy, OH, USA), with the final temperature maintained below 4 °C. The beef patties were shaped using a handheld patty maker (Ministeak burger maker, O.L. Smith Co. Ltd., Saline, Italy), packaged in polyethylene bags, and stored at 4 °C.

### 2.3. Proximate Composition of Raw Beef Patties

The moisture content in raw beef patties was determined following the described methodologies [25]. Protein content was measured using a LECO FP-200 Nitrogen Determinator (Leco Corp., St Joseph, MI, USA), while fat content was assessed according to the method described by Bligh and Dyer [26]. All determinations were performed in triplicate.

### 2.4. Microbiological Analysis

Each raw beef patty sample was collected under aseptic conditions and mixed with buffered peptone water (Panreac, Darmstadt, Germany). To determine the TVC, Plate Count Agar (PCA; Panreac) was employed and incubated at 37 °C for 48 h. Enumeration of the LAB was performed using De Man, Rogosa, and Sharpe agar (MRS; Merck, Darmstadt, Germany), with an incubation period of 48 h at 37 °C. For Enterobacteriaceae enumeration, Violet Red Bile Glucose Agar (VRBG; Panreac) with a double layer was used and incubated at 37 °C for 24 h. Microbiological analyses were conducted in duplicate, and microbial counts were expressed as logarithms of colony-forming units per gram (log CFU/g). Additionally, the presence of *Salmonella* sp. was assessed in 25 g of the beef patties. For this purpose, a 25 g sample was mixed with 225 mL of sterile buffered peptone water solution (0.1%, Panreac) for bacterial prepropagation. Selective proliferation of bacteria was carried out using Rappaport-Vassiliadis Soy Broth medium (Merck) and bacterial isolation was performed using Hektoen (Merck KGaA, Darmstadt, Germany) and Xylose Lysine Deoxycholate (XLD; Merck) agars. Previously, the microbiological characterization of CF was conducted, and the results are as follows: TVC: 6.83 log CFU/g; Enterobacteriaceae: <1.6 log CFU/g; LAB: 2.71 log CFU; *Salmonella* sp.: absence; and *Listeria monocytogenes*: absence.

### 2.5. Cooking Process

The patties were cooked on an electric grill (JATA GR3000, Navarra, Spain), preheated to 180 °C, until they reached an internal temperature of 72 °C in their geometric center. To ensure uniform cooking, the patties were turned over at 1 min intervals. After cooking, they were allowed to cool to 25 °C.

### 2.6. Sensorial Analysis

Sensory evaluation was conducted on the cooked beef patties (as previously described) the day following their production. A panel of 16 semi-trained evaluators (members of different food research teams), well acquainted with the product and possessing prior experience in sensory evaluation of meat products, participated in the assessment. The samples were served in disposable plastic containers labeled with 3-digit random numbers. The panelists evaluated the appearance, color, texture, taste, and aroma of beef patties. During the sensory tests, water and unsalted crackers were provided to allow panelists to cleanse their palates between samples. The sensory evaluation was made on a 10 cm structured line, ranging from 0 to 10, where 0 represents the lowest intensity of each attribute and 10 represents the highest intensity. A purchase intention test was also performed using a 10 cm structured line scale, featuring the terms “I definitely would not buy” and “I definitely would buy” at opposite ends of the scale. Subsequently, each point on the scale was converted into a numerical value.

### 2.7. Technological Properties

The technological properties of beef patties were assessed both in raw and cooked beef patties, aiming to analyze their technological properties and the effects of the cooking process on these properties.

#### 2.7.1. Cooking Yield: Weight Loss and Diameter Reduction

Three beef patties of each formulation were weighed both before and after cooking to determine the weight loss (*CL*) as follows [27]:

(1) $$CL\;\left(\%\right)=\frac{{weight}_{raw}-{weight}_{cooked}}{{weight}_{raw}}\times 100$$

Additionally, the diameter reduction (*DR*) of the beef patties was measured using a caliper, which was based on the difference in the larger diameter of patties before and after cooking. This reduction was calculated as follows [27]:

(2) $$DR\;\left(\%\right)=\frac{{diameter}_{raw}-{diameter}_{cooked}}{{diameter}_{raw}}\times 100$$

#### 2.7.2. pH, Color Parameters, and Kramer Shear Force

The pH of both raw and cooked beef patties was determined in triplicate. To this end, 10 g of each sample was homogenized in 100 mL of distilled water at 25 °C. This measurement was conducted using a pH meter (model 827, pH Lab Methrom, Herisau, Switzerland) previously calibrated with standard buffers (pH = 4.00; pH = 7.00 at 25 °C).

The color values (L*: lightness, a*: redness, and b*: yellowness) were assessed on the surface of both raw and cooked beef patties. For each treatment, five determinations were made. A portable colorimeter (Konica Minolta CR-400, Tokyo, Japan) was employed under D65 illuminant and 10° standard observer with an 8 mm aperture. The beef patties were exposed to atmospheric air for 10 min before color measurement. The instrument was calibrated using a white standard plate before color readings.

The Kramer Shear Force (KSF) test was performed on both raw and cooked beef patties using a miniature Kramer (HDP/MK05) cell in a TA-XTplus Texture Analyzer (Stable Micro Systems Ltd., Godalming, UK) equipped with a 25 kg load cell. A mini 5-bladed head was used to conduct the shearing test. Samples measuring 2 × 2 cm were cut from two patties, weighed accurately, and placed into the cell at room temperature. A force was applied to a compression distance of 20 mm at 0.8 mm/s crosshead. KSF values were calculated as the maximum force per gram of sample (N/g). Measurements were carried out five times.

### 2.8. Statistical Analysis

The entire experiment was repeated twice. Analysis of variance (ANOVA) was performed using SPSS 26.0 (SPSS Inc., Chicago, IL, USA) to investigate the impact of CF addition on the composition, microbiological, sensorial, and technological properties of beef patties. A completely randomized design was employed, with treatment groups (Control, CF5.0, CF7.5, and CF10.0) treated as fixed effects, and the two replications treated as random. Furthermore, for technological properties (pH, color, and KSF), treatment groups (Control, CF5.0, CF7.5, and CF10.0) and the cooking process (raw and cooked) were considered as fixed effects, with the two replications considered as random effects. Means were compared using Tukey’s HSD test, and statistical significance was deemed present when *p* < 0.05.

## 3. Results and Discussion

### 3.1. Proximate Composition of Raw Beef Patties

The proximate composition of raw beef patties was conditioned to the incorporation of CF as a lean meat replacer (Table 1). However, the addition of CF had a significant impact (*p* < 0.05) only on moisture and protein content. Moisture values ranged between 62.87 and 68.05 g/100 g, and the control treatment exhibited the highest (*p* < 0.05) moisture values, while the CF10.0 treatment showed the lowest (*p* < 0.05). As expected, the control treatment exhibited lower (*p* < 0.05) protein content. However, the addition of CF significantly increased this content to values between 21.82% and 23.68% (CF5.0 < CF7.5 < CF10.0). In contrast, the fat content was not significantly affected by the incorporation of CF as a lean meat replacer, with all treatments maintaining fat levels around 9%. This is likely because beef meat and cricket flour had similar fat content while differing in protein content. This is a noteworthy observation because it indicates that the addition of CF at these levels can result in meat products with increased protein levels while preserving fat content.

### 3.2. Microbiological Analysis

The microbial counts of beef patties are summarized in Table 2. The incorporation of CF resulted in higher TVC (*p* < 0.05) in all treatments (Table 2). In terms of LAB counts, the values ranged from 3.95 to 4.37 log CFU/g. CF5.0 and CF7.5 exhibited similar (*p* > 0.05) LAB counts to the control (Table 1). Additionally, when comparing treatments with CF, no significant differences were found in LAB counts (Table 2). The elevated TVC and LAB counts in fresh meat products like beef patties with CF as a lean meat replacer could potentially result in reduced shelf life. However, it is crucial to note that these meat products are intended to be consumed in a cooked form, reaching an internal temperature of at least 72 °C in their geometrical center, which effectively eliminates the vegetative microbial load and makes them safe for consumption [28].

Enterobacteriaceae counts ranged between 1.73 and 1.94 log CFU/g, with no significant differences (*p* > 0.05) observed among treatments. Furthermore, Salmonella sp. was not detected in any of the beef patty samples. These results indicated that the production of these beef patties adhered to good hygiene and sanitary conditions and practices. Additionally, the microbiological properties of beef patties with CF addition align with the microbiological profile of dried crickets, as reported by Ververis et al. [29].

The microbiological properties of foods containing edible insects are of significant concern, including meat products, because insects are typically consumed with their digestive tracts regardless of the form of incorporation (whole or in powdered forms) [29]. Various processing steps can be employed to improve the microbiological profile of crickets when used as an ingredient in meat products. These steps may include heat treatments such as autoclaving, steaming, and boiling, as well as different drying methods like oven-drying, solar-drying, microwave-drying, spray-drying, and toasting. These methods can be applied individually or in combination to enhance the safety of using insects as an ingredient [29].

### 3.3. Sensory Characteristics

**Figure 1:**
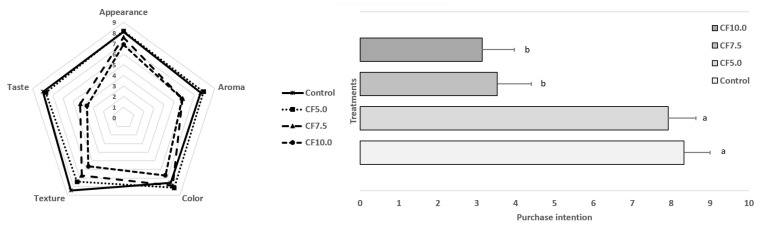
Sensory scores and purchase intention of beef patties. Different letters show significant differences among treatments (*p* < 0.05). Error bars indicate the standard deviation of the mean. Treatments: Control: no CF addition; CF5.0: addition of 5.0% CF; CF7.5: addition of 7.5% CF; and CF10.0: addition of 10.0% CF.

The results of the sensory analysis of the beef patties are summarized in Figure 1. The incorporation of CF as a lean meat replacer in beef patties had varying effects on sensory scores depending on the level of incorporation. However, the CF5.0 treatment exhibited similar (*p* > 0.05) sensory scores and purchase intention to the control (Figure 1). Generally, the addition of CF did not significantly affect (*p* > 0.05) the appearance, aroma, and color attributes of the beef patties. However, the texture, taste, and purchase intention attributes were notably impacted (*p* < 0.05) by the addition of CF (CF7.5 and CF10.0) (Figure 1). The popular reception of insects as food encompasses several dimensions that affect the willingness to eat, including psychological, social, cultural, contextual, ecological, and practical factors such as price, availability, and taste [30]. In this context, the lower sensory scores of beef patties with higher concentrations of insect protein may be linked to food neophobia, feelings of disgust, unfamiliarity with insect-based foods, and other nutritional and environmental psychological factors [31,32]. The superior sensory properties of the CF5.0 treatment may be attributed to the fact that these beef patties were more similar to the control group. According to McClements [33], consumers often prefer to eat insects when they are disguised in foods. Furthermore, lower (*p* < 0.05) scores for taste in beef patties with CF added at levels exceeding 5.0% could potentially be attributed to the bitter taste associated with higher levels of CF incorporation, as reported in other studies involving meat products [12,15]. The lower scores for texture and taste also had an adverse impact on purchase intention attributes, with CF7.5 and CF10.0 treatments scoring lower (*p* < 0.05) compared to the control and CF5.0 (Figure 1). Beef patties with high levels of CF incorporation (CF7.5 and CF10.0) may require interventions concerning taste to eliminate the bitter aftertaste of the product.

### 3.4. Technological Properties

#### 3.4.1. Cooking Yield: Weight Loss and Diameter Reduction

**Figure 2:**
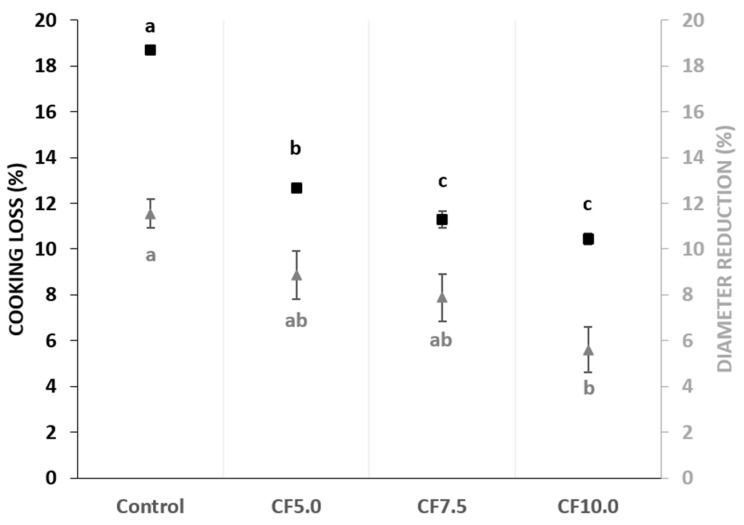
Cooking loss (■) and diameter reduction (▲) of beef patties. Different letters show significant differences among treatments (*p* < 0.05). Error bars indicate the standard deviation of the mean. Treatments: Control: no CF addition; CF5.0: addition of 5.0% CF; CF7.5: addition of 7.5% CF; and CF10.0: addition of 10.0% CF.

Both cooking loss and diameter reduction are critical technological parameters related to the WHC and OHC of proteins during the cooking process of beef patties [34]. The cooking loss of the beef patties, which ranged from 10.46% to 18.68%, was significantly higher in the control (*p* < 0.05) compared to the treatments with CF, with CF7.5 and CF10.0 presenting the lowest (*p* < 0.05) values (Figure 2). This reduction in cooking loss in beef patties is attributed to the beneficial WHC and OHC properties of CF, and the effect becomes more pronounced with increasing levels of CF. This aligns with the findings of Gomes Martins et al. [31], who reported that the cooking yield increases in beef patties with higher levels of *Gryllus assimilis* flour addition. Additionally, similar findings have been reported in other studies, where the partial addition of CF resulted in decreased cooking loss in cooked meat products [12,13].

The values of diameter reduction ranged from 5.60% to 11.55% (Figure 2). Changes in the beef patty diameter are significant and can be influenced by the inclusion of new ingredients [35]. The addition of CF as a lean meat replacer at high levels (CF10.0) in beef patties led to lower (*p* < 0.05) diameter reduction values when compared to the control treatment. However, no significant differences were observed between CF5.0, CF7.5, and the control (Figure 2). There is a direct relationship between cooking loss and diameter reduction values because the reduction in diameter of beef patties results from protein denaturation during heat treatment, leading to increased water and fat loss [36]. Higher diameter reductions, ranging between 14.06% and 18.55%, were observed in beef patties with *G. assimilis* addition [31]. However, the addition of insect flour resulted in a lower diameter reduction when compared to the standard formulation [31]. Other studies have reported diameter reduction values ranging from 14% to nearly 25% in beef and pork patties [36,37].

#### 3.4.2. pH, Color Parameters, and Kramer Shear Force

**Figure 3:**
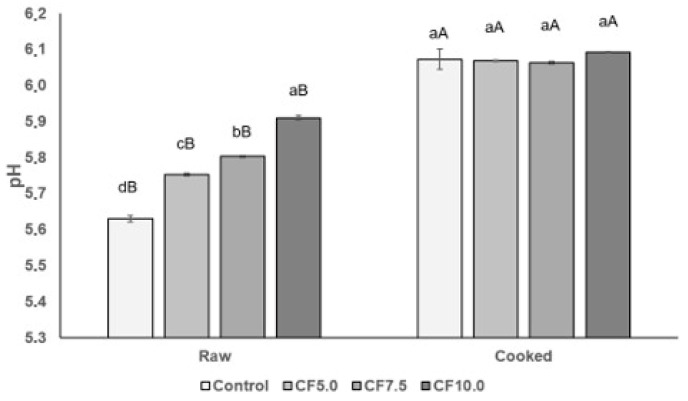
pH values of both raw and cooked beef patties. Different lowercase letters show significant differences among treatments (*p* < 0.05) and different uppercase letters show significant differences between raw and cooked (*p* < 0.05). Error bars indicate the standard deviation of the mean. Treatments: Control: no CF addition; CF5.0: addition of 5.0% CF; CF7.5: addition of 7.5% CF; and CF10.0: addition of 10.0% CF.

The pH values of all raw beef patties fell within the range of 5.60 to 5.91 (Figure 3), and they were significantly influenced (*p* < 0.05) by the addition of CF. The pH values of raw beef patties with CF incorporation were higher than those of the control (*p* < 0.05), which may be attributed to the higher pH value of CF (6.41) in comparison to that of lean meat [11]. Higher pH values were also observed in beef patties with *G. assimilis* flour addition, ranging between 6.07 and 6.17, compared to the control (5.98). This observation was attributed to the high pH of insect flour (6.55) [31]. This observation indicates the significance of pH in raw beef patties, as it can influence the growth of spoilage and pathogenic bacteria, subsequently affecting the shelf life and security of the product. During the cooking process, the pH increased significantly (*p* < 0.05) in all beef patties. However, all patties exhibited similar (*p* > 0.05) pH values after cooking (Figure 3), indicating that differences in the taste attribute of meat products with CF incorporation (Figure 1) are not caused by an acidic taste in the final product. While other studies have reported an increase in pH values in emulsified cooked meat products with *Tenebrio molitor* addition [38,39], the extent of this increase depends on the pH of the edible insect used. However, another study found that the pH values of frankfurter sausages were not affected by the addition of CF [12]. Nevertheless, the pH values of both raw and cooked beef patties were consistent with those reported in other studies. Parvin et al. [40] reported pH values ranging from 5.6 to 6.0 in raw beef patties and from 6.0 to 6.14 in cooked beef patties with nutmeg extract addition.

**Figure 4:**
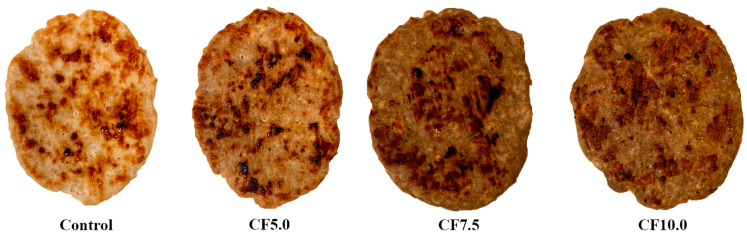
Typical appearance of cooked beef patties. Treatments: Control: no CF addition; CF5.0: addition of 5.0% CF; CF7.5: addition of 7.5% CF; and CF10.0: addition of 10.0% CF.

Color is a crucial attribute in meat products, as it is directly linked to consumer acceptance. According to Table 3, the addition of CF had a significant influence (*p* < 0.05) on the lightness parameter in both raw and cooked beef patties. The L* values ranged from 47.71 to 61.05, and the incorporation of CF as a lean meat replacer reduced lightness, resulting in darker raw beef patties (*p* < 0.05). However, when comparing the treatments with CF, the incorporation of 5.0% CF (CF5.0) produced raw beef patties with higher (*p* < 0.05) L* values than CF7.5 and CF10.0. The cooking process significantly decreased (*p* < 0.05) L* values in the control and CF5.0 (Table 3). No differences (*p* > 0.05) were observed between L* values in raw and cooked beef patties in treatments CF7.5 and CF10.0. Additionally, no significant differences (*p* > 0.05) in lightness were found between cooked control and CF5.0 treatments, but a significant decrease (*p* < 0.05) was observed in cooked CF7.5 and CF10.0 treatments (Table 3). This observation is also supported by the visual appearance of cooked beef patties (Figure 4). Other studies have reported that lightness decreases as the level of edible insects increases in cooked meat products [12,13,16,24,41].

According to Table 3, the a* values of raw beef patties ranged from 2.78 to 3.62. Although there is no clear significant trend, it appears that, in general, the addition of CF decreases the a* values of beef patties. The cooking process increased (*p* < 0.05) a* values in all treatments, but no differences (*p* > 0.05) were found among all cooked beef patties. Yellowness values ranged from 6.94 to 9.80 in raw beef patties, with the treatments with CF addition having lower (*p* < 0.05) b* values than the control. The cooking process significantly increased (*p* < 0.05) b* values in the control, but it decreased (*p* < 0.05) this parameter in CF5.0, CF7.5, and CF10.0. Consequently, the cooked control exhibited the highest (*p* < 0.05) b* values, while cooked CF7.5 and CF10.0 had the lowest (*p* < 0.05) values. Based on the instrumental color results, the reformulated beef patties with CF as a lean meat replacer appeared more brownish compared to the control, which could be attributed to the brownish color of CF [12]. It is worth noting that, despite the changes in the instrumental color of beef patties with CF incorporation, the products received sensory scores no lower than the control for this parameter (Figure 1).

**Figure 5:**
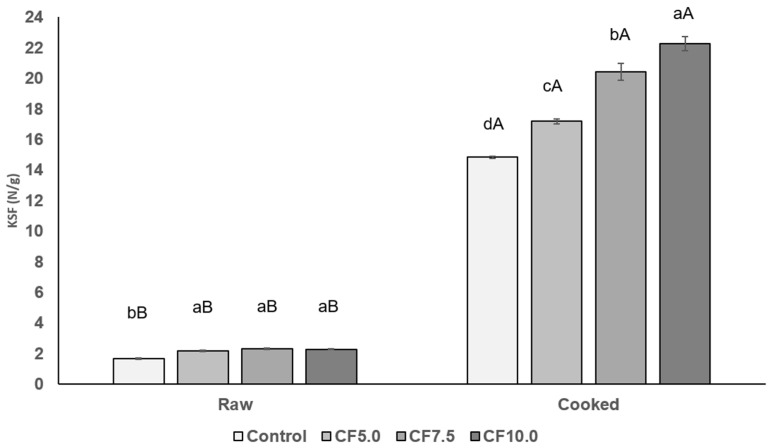
Kramer Shear Force (N/g) values of both raw and cooked beef patties. Different lowercase letters show significant differences among treatments (*p* < 0.05) and different uppercase letters show significant differences between raw and cooked (*p* < 0.05). Error bars indicate the standard deviation of the mean. Treatments: Control: no CF addition; CF5.0: addition of 5.0% CF; CF7.5: addition of 7.5% CF; and CF10.0: addition of 10.0% CF.

The values of KSF in raw beef patties ranged from 1.67 N/g to 2.34 N/g (Figure 5). The incorporation of CF produced harder raw beef patties with higher (*p* < 0.05) KSF values, regardless of the amount of CF added. On the other hand, another study reported that beef patties incorporating *G. assimilis* flour resulted in a softer texture [31]. The cooking process significantly increased the KSF values in all treatments (*p* < 0.05). Additionally, cooked beef patties with CF were significantly harder than the control (*p* < 0.05), and this effect became more pronounced as more CF was added (Figure 5). These results may be related to texture attributes in the sensory analysis (Figure 1), indicating that this treatment had a hard texture also noted by the panelists. The higher (*p* < 0.05) KSF values in raw and cooked beef patties with CF addition indicate a firmer texture compared to the control treatment. The addition of CF protein forms a denser protein matrix [42], resulting in a firmer texture. This was also observed in emulsified cooked sausages [11,43]. However, the replacement of lean meat with edible insects at the levels performed in this study resulted in a final product with a similar firmness to other patties reported in the literature [44,45].

## 4. Conclusions

The reformulation procedure using CF as a lean meat replacer in beef patties resulted in products with higher protein content. Despite beef patties with CF showing high TVC and LAB counts, the levels of Enterobacteriaceae and *Salmonella* sp. indicated that good hygiene and sanitary practices were followed during their production. Sensory characteristics and purchase intention were affected by the level of CF addition. Incorporating up to 5.0% CF resulted in beef patties with similar sensory attributes and purchase intention as traditional beef patties (control). However, when CF levels were between 7.5% and 10.0%, there were changes in sensory properties. The use of CF reduced cooking loss and diameter reduction while increasing browning and hardness. Generally, after cooking, beef patties made with CF underwent changes to their technological properties identical to the products made entirely with raw beef meat. However, cooked beef patties made with CF were harder and more brownish than the control. This study indicates that, under similar conditions, the optimal level of CF incorporation as a lean meat replacer in beef patties is 5.0%. Despite the promising results in obtaining beef patties with high protein content and improved technological properties, some negative aspects, particularly in terms of taste in sensory analysis and microbiological parameters, have also been identified. Therefore, further studies are being conducted to address and eliminate these drawbacks. Nevertheless, the results of this study offer valuable research material for the potential industrial application of CF as an alternative protein source. This avenue can be considered a promising direction for the development of healthier and more environmentally friendly meat products in the future.

## Figures and Tables

**Table 1:** Proximate composition (moisture, protein, and fat (g/100 g)) of beef patties.

Parameter	Treatments			
	Control	CF5.0	CF7.5	CF10.0
Moisture	68.05 ± 0.20 ^a^	65.39 ± 0.03 ^b^	64.94 ± 0.04 ^b^	62.87 ± 0.17 ^c^
Protein	19.53 ± 0.03 ^d^	21.82 ± 0.08 ^c^	22.70 ± 0.14 ^b^	23.68 ± 0.04 ^a^
Fat	8.54 ± 0.16 ^a^	8.79 ± 0.20 ^a^	9.09 ± 0.21 ^a^	9.20 ± 0.14 ^a^

Means ± standard deviation. Different superscript lowercase letters (a–d) in a row indicate statistically significant differences (*p* < 0.05). Treatments: Control: no CF addition; CF5.0: addition of 5.0% CF; CF7.5: addition of 7.5% CF; and CF10.0: addition of 10.0% CF.

**Table 2:** Microbiological analysis (log CFU/g) of raw beef patties.

Microorganisms	Treatments			
	Control	CF5.0	CF7.5	CF10.0
Total viable count	4.59 ± 0.12 ^c^	6.36 ± 0.07 ^ab^	6.23 ± 0.00 ^b^	6.62 ± 0.00 ^a^
Lactic acid bacteria	3.95 ± 0.02 ^b^	4.24 ± 0.17 ^ab^	4.21 ± 0.02 ^ab^	4.37 ± 0.03 ^a^
Enterobacteriaceae	1.73 ± 0.16 ^a^	1.77 ± 0.10 ^a^	1.75 ± 0.21 ^a^	1.94 ± 0.33 ^a^

Means ± standard deviation. Different superscript lowercase letters (a–c) in a row indicate statistically significant differences (*p* < 0.05). Treatments: Control: no CF addition; CF5.0: addition of 5.0% CF; CF7.5: addition of 7.5% CF; and CF10.0: addition of 10.0% CF.

**Table 3:** Color parameters (L*, a*, and b*) of raw and cooked beef patties.

Parameters	Cooking	Treatments			
		Control	CF5.0	CF7.5	CF10.0
Lightness	Raw	61.05 ± 1.10 ^a,A^	52.99 ± 1.35 ^b,A^	49.75 ± 1.47 ^c,A^	47.71 ± 1.60 ^c,A^
	Cooked	55.97 ± 2.84 ^a,B^	50.32 ± 1.78 ^b,B^	50.11 ± 2.42 ^b,A^	48.40 ± 0.48 ^b,A^
Redness	Raw	3.62 ± 0.34 ^a,B^	3.08 ± 0.37 ^b,B^	3.20 ± 0.16 ^a,b,B^	2.78 ± 0.31 ^b,B^
	Cooked	5.26 ± 1.06 ^a,A^	5.15 ± 0.54 ^a,A^	4.55 ± 0.71 ^a,A^	4.28 ± 0.30 ^a,A^
Yellowness	Raw	9.80 ± 0.73 ^a,B^	7.74 ± 0.84 ^b,A^	7.63 ± 0.75 ^b,A^	6.94 ± 0.94 ^b,A^
	Cooked	11.77 ± 1.21 ^a,A^	6.61 ± 0.68 ^b,B^	4.71 ± 0.75 ^c,B^	4.19 ± 0.89 ^c,B^

Means ± standard deviation. Different superscript lowercase letters (a–c) in a row indicate statistically significant differences (*p* < 0.05). Different superscript uppercase letters (A,B) in same column (corresponding to the same parameter) indicate statistically significant differences (*p* < 0.05). Treatments: Control: No CF addition; CF5.0: addition of 5.0% CF; CF7.5: addition of 7.5% CF; and CF10.0: addition of 10.0% CF.

## Data Availability

Data is contained within the article.
